# Inhibition of the JAK2/STAT3 Pathway Reduces Gastric Cancer Growth *In Vitro* and *In Vivo*

**DOI:** 10.1371/journal.pone.0095993

**Published:** 2014-05-07

**Authors:** Louise M. Judd, Treve R. Menheniott, Hui Ling, Cameron B. Jackson, Meegan Howlett, Anastasia Kalantzis, Waldemar Priebe, Andrew S. Giraud

**Affiliations:** 1 Infection and Immunity Division, Murdoch Children’s Research Institute, Parkville, Victoria, Australia; 2 Department of Experimental Therapeutics, The University of Texas MD Anderson Cancer Center, Houston, Texas, United States of America; 3 Department of Pathology and Cancer Research Institute, University of South China, Hengyang, Hunan, China; Vanderbilt University School of Medicine, United States of America

## Abstract

Signal Transducer and Activator of Transcription-3 (STAT3) is constitutively activated in many cancers where it promotes growth, inflammation, angiogenesis and inhibits apoptosis. We have shown that STAT3 is constitutively activated in human gastric cancer, and that chronic IL-11-driven STAT3 transcriptional activity induces gastric tumourigenesis in the gp130^757FF^ mouse model of gastric cancer development. Here we show that treatment of human AGS gastric cancer cells with the Janus Kinase (JAK) inhibitor WP1066 dose-, and time-dependently inhibits STAT3 phosphorylation, in conjunction with reduced JAK2 phosphorylation, reduced proliferation and increased apoptosis. In addition, application of intraperitoneal WP1066 for 2 weeks, reduced gastric tumour volume by 50% in the gp130^757FF^ mouse coincident with reduced JAK2 and STAT3 activation compared with vehicle-treated, littermate controls. Gastric tumours from WP1066- treated mice had reduced polymorphonuclear inflammation, coincident with inhibition of numerous proinflammatory cytokines including IL-11, IL-6 and IL-1β, as well as the growth factors Reg1 and amphiregulin. These results show that WP1066 can block proliferation, reduce inflammation and induce apoptosis in gastric tumour cells by inhibiting STAT3 phosphorylation, and that many cytokines and growth factors that promote gastric tumour growth are regulated by STAT3-dependent mechanisms. WP1066 may form the basis for future therapeutics against gastric cancer.

## Introduction

Of the seven Signal Transducer and Activator of Transcription (STAT) family members, STAT3 has been most consistently implicated in a range of common cancers in humans, including; lung, breast, ovarian, prostate, and colon [1], [2], [3], [4]. This is also true in human gastric cancer [5], [6], [7] in which STAT3 activation by chronic phosphorylation at tyrosine (Y) reside 705 has been linked to increased growth, angiogenesis, invasion and metastasis of the primary cancer [6], [7], [8]. Thus, inhibition of STAT3 transcriptional activity in human gastric cancer may provide a possible means of reducing the high morbidity and prolong life amongst gastric cancer patients worldwide.

In the absence of functional mutations in the STAT3 gene, aberrant STAT3 activity is induced by persistent activity from upstream tyrosine kinases, and/or by unscheduled- or over-expression of stimulatory ligands [9], [10], [11]. This is clearly exemplified in the gp130^757F/F^ mouse model of gastric cancer development, in which a Phe for Tyr substitution at the 757 position on the intracellular arm of the IL-6 family signalling receptor gp130 simultaneously prevents SHP2 and SOCS3 binding, resulting in inhibition of ras/MAP kinase signal transduction, and hyperactivation of STAT3 by constitutive phosphorylation [23], [24], [26]. Recently we and others have shown that in gastric tumours, significant increases in transcription coincide with increased expression of the gp130 ligand IL-11 in human gastric cancer and mouse models of this disease [5], [12], [13]. In the latter IL-6 is dispensable, but IL-11 is absolutely required for tumourigenesis [12], [13]. Additionally, IL-11/STAT3 has been shown to be an important driver of atrophic gastritis, the first precancerous lesion of the stomach following chronic infection by the bacterium *Helicobacter pylori* [14].

To date, numerous kinases have been reported to induce STAT3 activity following ligand/receptor binding, however only JAK1 and JAK2 account for STAT3 phosphorylation upon docking with IL-11/gp130 receptor complex [15] and of these IL-11/gp130 preferentially binds JAK2 [16]. These observations suggest that JAK2 and STAT3 present promising targets for designing therapeutic antagonists to suppress IL-11/STAT3 signalling in human gastric cancer.

Recently the caffeic acid derivative WP1066, structurally related to the low potency tyrosine kinase inhibitor AG490, has been shown to be a highly potent inhibitor of the JAK2/STAT3 pathway in transformed brain glioma [17] and renal carcinoma [18] cell lines, leading to growth inhibition and induction of classical pro-apoptotic pathways. In addition, WP1066 is effective *in vivo* against highly malignant melanomas and leukemias that are positive for the JAK2-V617F+ mutation, which promotes constitutive JAK2 kinase activation [19], [20], [21], [22]. Unpublished studies indicate that WP1066 is not an ATP-competitive inhibitor, and can block expression of phosphorylated JAK2 and STAT3; in addition, p-STAT3-Y705 can be inhibited independently of the JAK2 status. Thus, WP1066 presents a unique opportunity to inhibit both p-JAK and p-STAT3, and subsequently potently block JAK2/STAT3 signalling pathway and STAT3 transcriptional activity.

To test the idea of dual blockade of JAK2 and STAT3 activation in the stomach and subsequent gastric cancer development, we have used both *in vitro* and *in vivo* approaches to assess whether WP1066 can slow or block gastric tumour growth through inhibition of JAK2/STAT3 activity, and other closely related oncogenic signalling pathways. Here we show that WP1066 effectively inhibits STAT3 phosphorylation, and induces apoptosis in a gastric cancer cell line, and that it can inhibit gastric tumour growth *in vivo* by blocking induction of key STAT3-regulated genes.

## Materials and Methods

### Preparation and Storage of Kinase Inhibitors

Inhibitor WP1066 was developed and synthesised by Waldemar Priebe and coworkers at the University of Texas MD Anderson Cancer Center, and current stock was supplied courtesy of Houston Pharmaceuticals Inc, Houston, Texas, USA. Stocks were resuspended in Hybri-Max DMSO and stored at −20°C. Stocks were single use only, and not re-frozen upon thawing.

### In vitro Culture

AGS cells (ATCC, Manassas VA, USA) cells were maintained in complete media containing RPMI+Glutamax (Gibco Life Sciences, Invitrogen OR, USA) media supplemented with 10% foetal bovine serum, 50 IU penicillin at 37°C in 5% CO_2_–95% air.

### Western Blotting

Protein extracts were prepared with either TRIzol reagent (Life Technologies, Vic, Australia) according to the manufacturer’s instructions and protein pellets were resuspended in 1% sodium dodecyl sulfate containing 2 mmol/l Na_3_VO_4_ or RIPA buffer. Aliquots (30 µg) were subjected to sodium dodecyl sulphate/polyacrylamide gel electrophoresis. Membranes were blocked and incubated at 4°C overnight in skim milk with the following antibodies; STAT3, pY(705)STAT3, ERK1/2, pT(202), Y(204)ERK1/2, AKT, pS(473)AKT, JAK2, pY(1007), Y(1008)JAK2 (Cell signaling, #9132, #9134S, #9131, #9102 #4377, #9272, #4058, #3752, #3751, #3229, #3771), GAPDH (Abcam #9485). Membranes were incubated with peroxide-conjugated secondary antibody (Dako, polyclonal swine anti rabbit, HRP conjugated, #P0399) and visualized by enhanced chemiluminescence (Amersham, Buckinghamshire, UK). For analysis bands were quantified using the Quantity One software system (Biorad) and phosphorylated proteins expressed as a proportion of GAPDH from a duplicate membrane. At least n = 8 samples were assessed by either treatment.

### Cell Counting by Haemocytometry

Cells were seeded at 5×10^4^ cells/ml in 24-well format in complete media and allowed to grow undisturbed for 24 hours. Cells were treated with the appropriate concentration of WP1066 or DMSO as control for 0–360 min. After treatment cells were dislodged by treatment with trypsin-EDTA 0.25% (Sigma), stained with trypan-blue 0.4% (Sigma) at a 1∶1 dilution for 5 minutes at 4°C, and counted on a haemocytometer.

### Carboxyfluorescein Diacetate Succinimidyl Ester (CFSE) Staining

To label AGS cells with CFSE, cells were dislodged by treatment with trypsin-EDTA 0.25% (Sigma) and resuspended in prewarmed PBS/0.1% BSA at a density of 1×10^6^ cells/ml. 10 mM CFSE dye (Invitrogen) was prepared as per manufacturer’s protocol, then 5 µl/ml was added and mixed thoroughly by inversion to ensure homogenous labelling of cells. Samples were incubated at 37°C for 10 minutes, quenched by addition of 5 volumes of ice-cold media and incubated for 5 minutes on ice. Samples were then washed 5 times in media and plated at 2×10^5^ cells/well (6-well dishes). A sample of cells was taken after plating and labelled with 100 µg/ml propidium iodide (PI) and analysed by flow cytometry for uniformity and intensity of labelling. Cells were then grown in complete media for 48 hours after which they were treated with or without appropriate WP1066 (5 µM) at 37°C with 5% CO_2_ 95% air for 18 hours. Cells were then trypsinised as above and resuspended in complete media, labelled with 100 µg/ml PI, and analysed by flow cytometry at 488 ηM. Data was recorded using the BD FACS diva (BD Biosiences) software package and analysed by Modfit LT software package (VSH).

### Annexin V Staining

AGS cells were grown in complete media at 2×10^5^ cells/well in 6-well plates with DMSO (carrier control), or WP1066 (5 µM), or etoposide (200 µM; positive control) at 37°C with 5% CO_2_ 95% air undisturbed for 24 hours. Cells were then treated as follows; washed in ice-cold PBS, trypsinised as above, and cell density determined by haemocytometry before resuspension in Annexin binding buffer to 1×10^6^ cells/ml. 100 µl of this preparation was taken, and incubated with 5 µl of Component A (Annexin Fluor 488 annexin V component, 25 mM HEPES, 140 mM NaCl, 1 mM EDTA, pH 7.4, 0.1% bovine serum albumin) and 1 µg/ml of PI for 15 minutes at room temperature in the dark. Samples were then mixed with 400 µl of Annexin binding buffer and analysed by flow cytometry. Appropriate controls +/− reaction components were prepared to adjust for bias in gating. Data was recorded using the BD FACS diva (BD Biosiences) software package.

### Ethics Statement

All animal experiments were performed in accordance with the Australian Code for the care and use of animals for scientific purposes (7^th^ edition), and after approval from the Murdoch Childrens Research Institute Animal Ethics Committee (application #A583). All efforts were made to minimize discomfort in these minimally invasive procedures.

### *In vivo* Experiments

gp130^757F/F^ mice have been previously described [23]. Briefly, discrete antral tumours develop by 4 weeks of age and grow rapidly until about 12 weeks. They recapitulate the developmental characteristics of intestinal-type gastric adenocarcinoma, including submucosal invasion but do not metastasise [24]. Animals were housed in an SPF facility at the Murdoch Children’s Research Institute and confirmed to be free of *Helicobacter pylori*. Three groups of mice were assessed for tumour development: 1) 8 week old gp130^757F/F^ mice that received no treatment (n = 10); 2) gp130^757F/F^ mice that received WP1066 from 8 to 10 weeks of age (n = 10); and 3) gp130^757F/F^ mice that received DMSO vehicle from 8 to 10 weeks of age (n = 8). Prior to experimentation all animals were assessed for wellbeing, weighed and treatment volumes calculated accordingly. Control animals received equivalent volumes of DMSO vehicle to WP1066-treated animals. Mice received 2 initial intra-peritoneal injections of WP1066 at 10 mg/kg every 48 hrs to acclimatise them to the effects of treatment, then received 5 injections of WP1066 at 20 mg/kg every 48 hr to complete the 2 week regimen. Intra-peritoneal injection sites were alternated through four quadrants of the abdomen of mice. At the conclusion of the experiment, mice were euthanized, and stomachs resected for photography and tissue collection.

### Macroscopic and Histological Assessment

Stomachs were rapidly dissected along the lesser curvature, pinned out, photographed and fixed in 4% buffered paraformaldehyde before processing. Paraffin sections (4 µm) were stained with haematoxylin and eosin (H&E). Morphometric analysis was performed using open-source ImageJ software (http://rsb.info.nih.gov/ij/index.html). For area measurements, images of gastric mucosa were manually outlined with the software drawing tool and the quantitation program used to generate measurements.

### *In vivo* Immunohistochemistry & Quantification

Immunohistochemical analysis was performed with antibodies for Ki-67 (Pharmingen #550609) and activated caspase 3 (Cell Signalling Technology #9961). Antigen retrieval was performed by boiling sections for 30 minutes in 10 mM citric acid (pH 6.0). Staining was completed with appropriate species-specific biotinylated secondary antibodies (DAKO, Denmark), avidin and biotinylated horseradish peroxidase macromolecular complex (Vector Laboratories, Burlingame, CA), 3,3′-diaminobenzidine (Sigma, St Louis, MI) and counterstained with hematoxylin. For Ki-67 quantification, a blinded observer counted number of stained cells per gland in multiple sections per animal using ImageJ as before. Data was expressed as the number Ki-67 positive cells per gland. For activated caspase 3 quantification, a blinded observer counted the number of stained cells per area of mucosa in 4 random areas of well orientated antrum per animal using ImageJ as before. Data was expressed as the number activated caspase 3 positive cells per area of mucosa.

### Semi-quantitative Morphometric Analysis of Inflammation

Inflammation was assessed using microscopy in a blinded fashion on H&E-stained sections. Antral tumour tissues were analysed and a minimum of 3 strips per animal (n = 7) were given a semi-quantitative score according to the degree of inflammatory cells from minimum = 0 to maximum = 3. Lymphoplasmocytic and polymorphonuclear infiltrate were assessed independently. The mean values were then compared for statistical analysis.

### Real-Time (Q)-PCR Analysis

RNA was extracted with TRIzol reagent (Life Technologies, Vic, Australia) according to manufacturer’s instructions. Total RNA (3 µg) was reverse transcribed into cDNA using Moloney murine leukemia virus reverse transcriptase (Promega, Madison, WI) primed with 0.3 µg oligo (dT). Q-PCR primers were designed using PRIMER EXPRESS (Applied Biosystems) (Table 1). SYBR green chemistry (Applied Biosystems) was used with L32 as the normalizer. The PCR conditions were 95°C for 10 min, then 40 cycles of 95°C for 15 sec and 60°C for 15 sec; the reactions were run on an Applied Biosystems AB7500 RT PCR machine. Results were analysed using sequence detector software, and relative fold differences were determined using the ΔΔCt method as described by the manufacturer.

**Table 1:** Primer sequences used for mRNA expression analysis by QRT-PCR.

Gene	Forward Primer	Reverse Primer
IL-1α	AACCCATGATCTGGAAGAGACC	TGGTGCTGAGATAGTGTTTGTCC
IL-1β	CAGGCAGTATCACTCATTGTGG	GTGCAGTTGTCTAATGGGAACG
RegI	ATCGTTGAGTTGCATCCTAAGC	CAATAGGAGCTGTAGGCATTGG
IL-11	CTGCAAGCCCGACTGGAA	AGGCCAGGCGAGACATCA
IL-6	ACAAAGCCAGAGTCCTTCAGAGA	CTGTTAGGAGAGCATTGGAAATTG
TNFα	TATGTCTCAGCCTCTTCTCATTCC	ATGATCTGAGTGTGAGGGTCTGG
γ-IFN	TGCCACGGCACAGTCATT	CCAGTTCCTCCAGATATCCAAGA
COX-2	TGCCTCCCACTCCAGACTAGA	CAGCTCAGTTGAACGCCTTTT
Amphiregulin	CTATCTTTGTCTGCCATCATC	ACAGACGGACGACAGTACTACA
HB-EGF	AGGGGTTAGGGAAGAAGAGAGA	ACAGACGGACGACAGTACTACA
iNOS	GGCAGCCTGTGAGACCTTTG	TGAAGCGTTTCGGGATCTG

### Statistical Analysis

The data was expressed as mean ± standard error of the mean. Values obtained from quantitative analysis of gene expression or number of stained cells was compared between samples by ANOVA, and where a statistically significant difference was found individual groups were further analysed with the appropriate parametric or non-parametric statistic using the Sigmastat statistical package. Statistical significance was defined as p≤0.05.

## Results

### WP1066 Rapidly Suppresses Phosphorylated Y(705)STAT3 and Induces Phosphorylation of ERK1/2 in AGS Cells

The JAK-STAT pathway and the ERK1/2 pathway are the two major signal transduction pathways downstream of gp130. These two pathways have reciprocal and inverse regulatory effects on each other, best demonstrated by the negative regulation of pSTAT3 after ERK1/2 activation [47].

**Figure 1:**
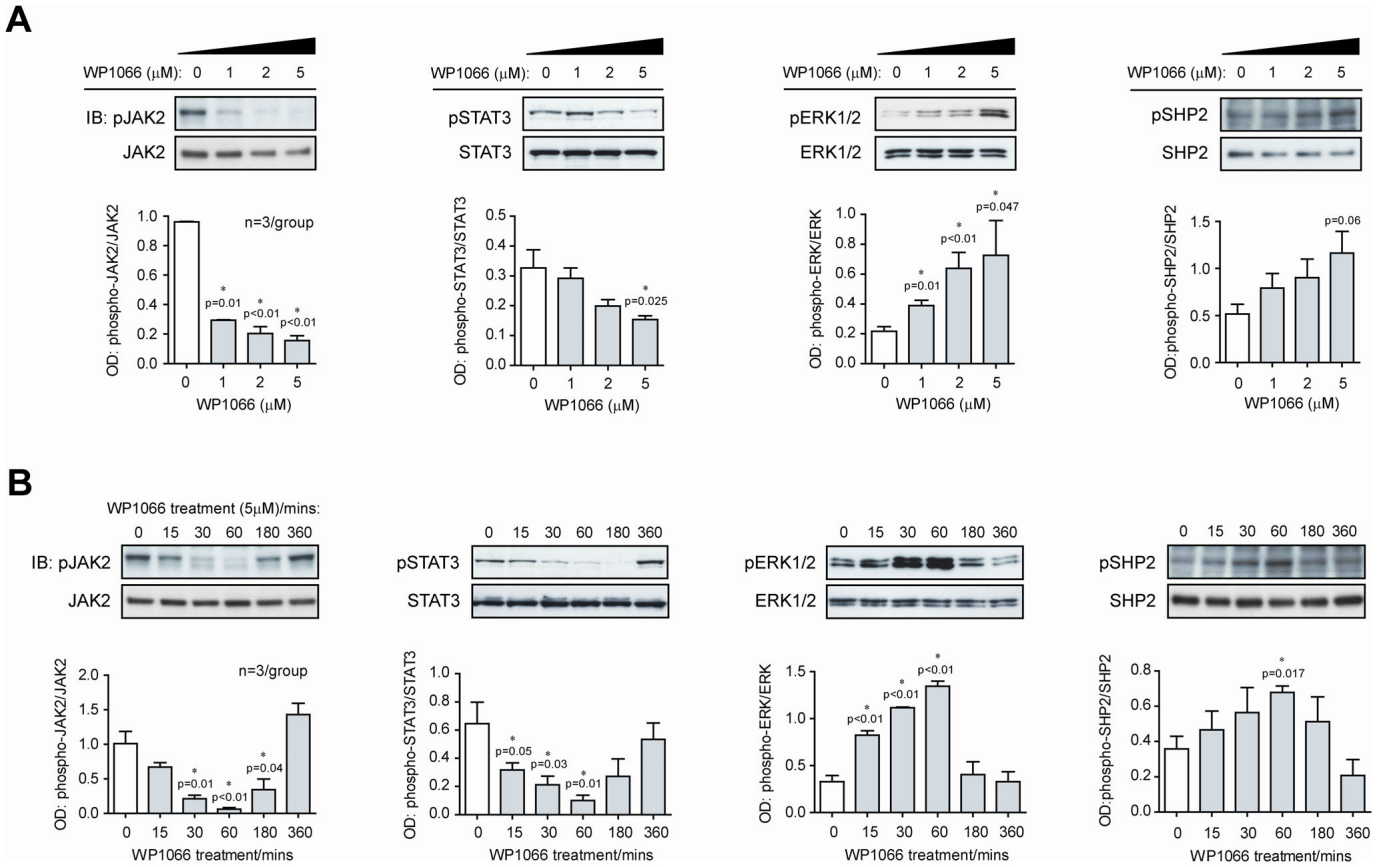
WP1066 modulates STAT3 and MAPK signalling endpoints in AGS cells. A. Dose-response: AGS cells were treated with WP1066 at 0, 1, 2 and 5 µM for 60 min and extracts immunoblotted with antibodies specific for (i) pJAK2 and JAK2; (ii) pSTAT3 and STAT3; (iii) pERK1/2 and ERK1/2; (iv) pSHP2 and SHP2. Data was also expressed as a ratio of the phosphorylated to total protein product in each case. The outcomes of 3 experiments are shown as mean OD ratios±standard error (SE). Statistical significance for each treatment compared to 0 µM WP1066 is shown if p<0.05. B. Time-course: AGS cells were treated as in Fig. 1A but with 5 µM WP1066 for 0, 15, 30, 60, and 180 min. Data was treated as for Fig. 1A.

WP1066 dose-response experiments were carried out by treating AGS cells with 0,1, 2 or 5 µM WP1066 for 60 min and measuring phosphorylation of JAK2, STAT3, ERK1/2 and SHP-2. As expected, pJAK2 was strongly inhibited by WP1066 at all concentrations tested and by >80% at 5 µM. 5 µM WP1066 also gave maximum inhibition of pSTAT3 and reciprocal activation of pERK1/2 (Fig. 1A) with a reduction in total STAT3 at 5 µM, or higher concentrations (data not shown). Coincident with the increase in pERK, pSHP2 activation was dose-dependently enhanced after WP1066 administration (Fig. 1A).

For time-course studies, AGS cells were treated for 0–360 min with 5 µM WP1066 and phosphorylation of JAK2, STAT3, ERK1/2 and SHP-2 were analysed by Western blotting (Fig. 1B). WP1066 treatment resulted in a rapid inhibition of pJAK2, with a 75% fall by 30 min and a maximum 90% fall by 60 min. Thereafter the cells recovered and returned to basal JAK2 phosphorylation by 360 min. The pattern of decreased STAT3 activation mirrored that of JAK2 phosphorylation. In contrast, ERK1/2 phosphorylation was markedly increased in response to WP1066 treatment, with a 250% increase by 15 min, and a maximal 460% increase by 60 min, before returning to basal levels by 360 min. SHP2 activation closely followed changes in ERK expression with maximal induction at 60 min.

### WP1066 Inhibits AGS Growth through Suppression of Proliferation and Induction of Apoptosis

Activated STAT3 is an established driver of human gastric cancer cell growth and proliferation [5], and prolonged activation of ERK1/2 induces apoptosis of gastric epithelial cells [25]. Having demonstrated that WP1066 regulates STAT3 and ERK1/2 signalling in a reciprocal fashion, we tested whether it can also perturb cell growth and induce apoptosis of AGS cells.

**Figure 2:**
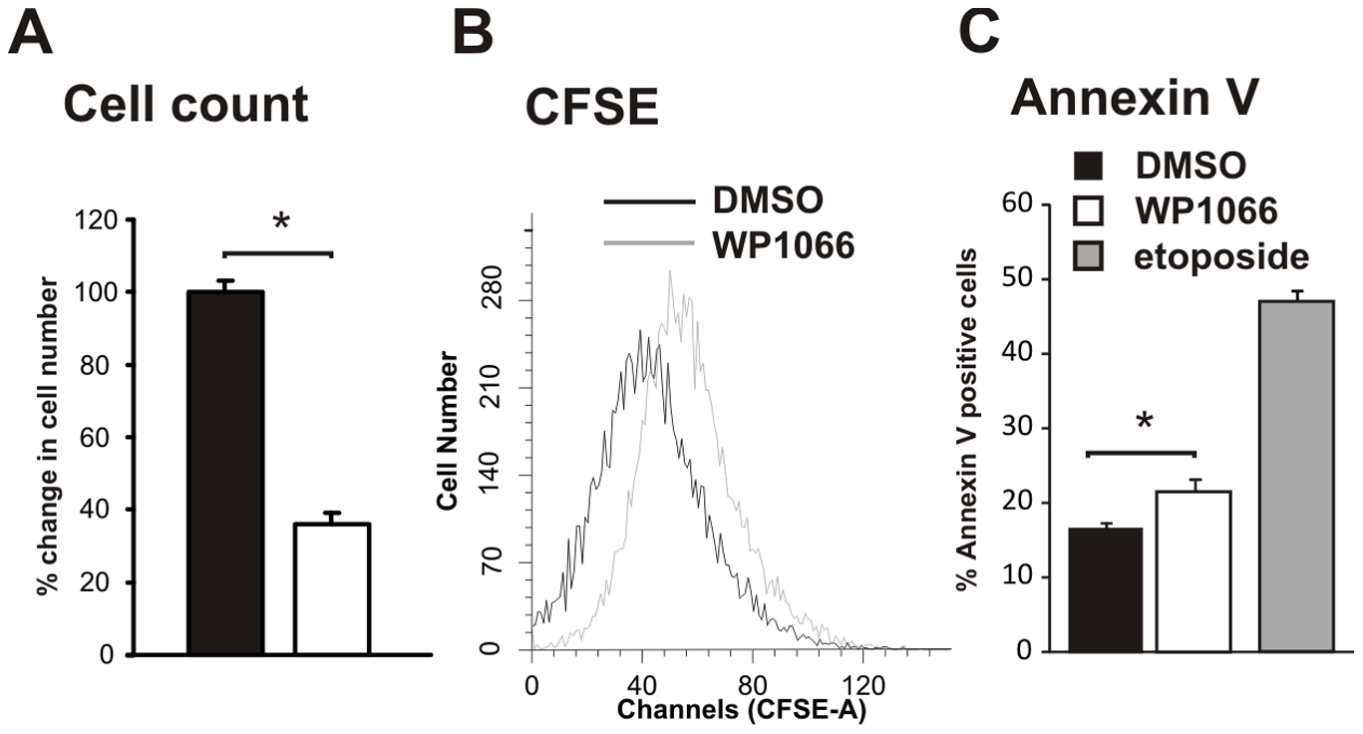
WP1066 inhibits proliferation and stimulates apoptosis of AGC cells. Proliferation: A. AGS cells were treated with WP1066 at 5 µM for 18 hr, stained with trypan blue and viable cells counted. Data are expressed as the percentage of viable cells compared to vehicle alone. N = 3, mean±SE. B. CFSE tracking was used to measure changes in AGS cell proliferation after treatment with WP 1066. The raw fluorescence data collected is shown compared to DMSO controls for a representative experiment. Apoptosis: C. AGS cells treated for 24 hr with DMSO (method control), WP1066 (5 µM) or etoposide (200 µM; positive control) and adherent cells were stained with Annexin V then counted manually. Data are expressed as fold change compared to DMSO vehicle from a representative experiment. *p = 0.047.

Treatment of AGS cells with 5 µM WP1066 (Fig. 2A) resulted in a substantial reduction in cell number relative to DMSO controls (DMSO; 100%±3.14 vs. WP1066; 36.02%±3.18, p<0.05). To determine if this reduction in cell number was due to altered cell proliferation or apoptosis, or a combination of both, CFSE and Annexin V staining was performed on treated cells. AGS cells treated with WP1066 were more intensely labelled with CFSE than those treated with DMSO, evidence of reduced number of cell divisions and therefore proliferation (Fig. 2B). Additionally, a larger proportion of AGS cells treated with WP1066 were labelled with Annexin V (Figure 2C) compared to cells treated with DMSO, demonstrating that the WP1066 also induced apoptosis in AGS cells (WP1066; 1.3 fold increase; p = 0.049).

### WP1066 Blocks Gastric Tumour Growth in gp130^757FF^ Mice by Suppression of Tumour Cell Proliferation and Enhancement of Apoptosis

**Figure 3:**
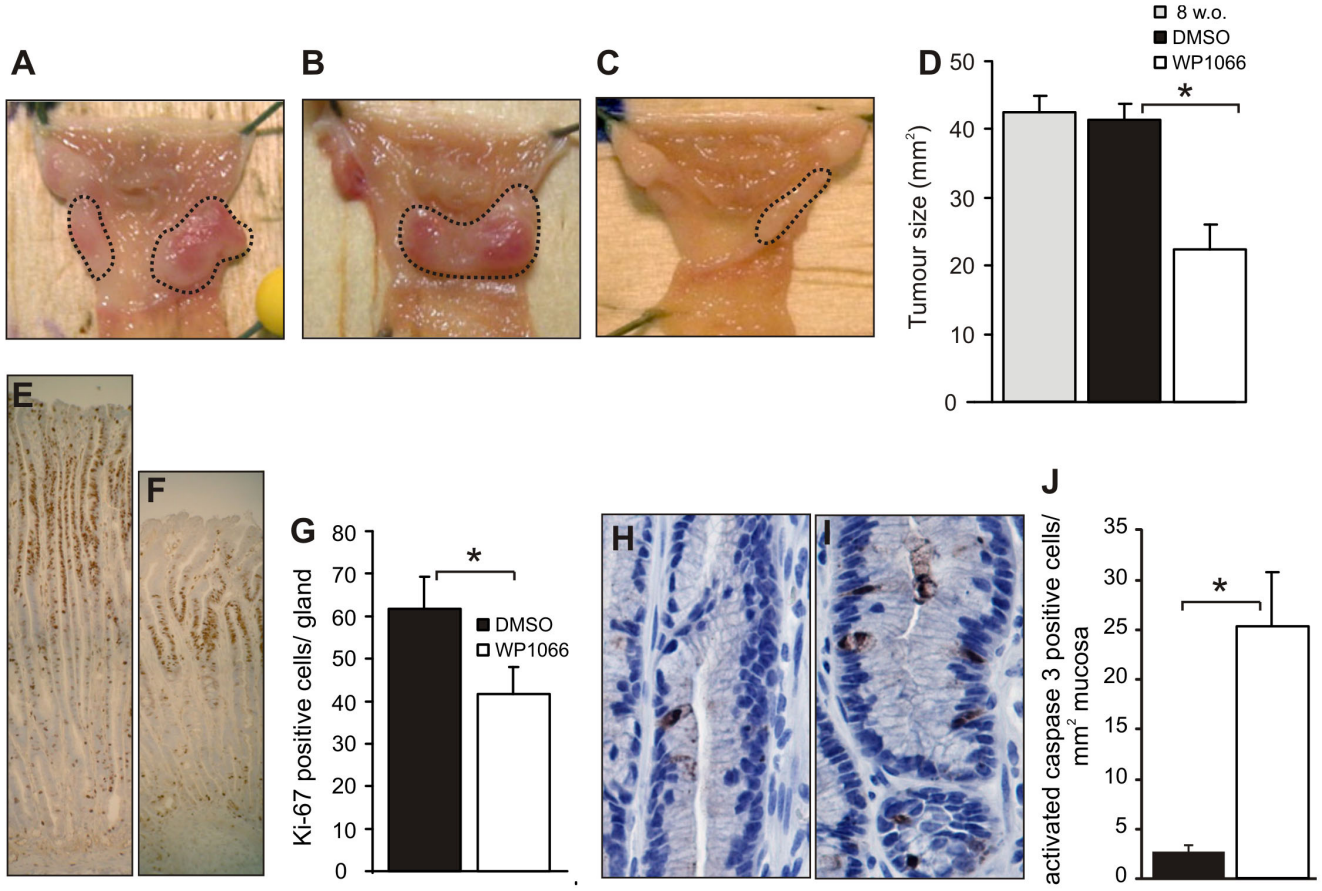
Treatment of gp130^757FF^ mice with WP1066 reduces tumour size. gp130^757FF^ mice (8 weeks old) were treated i.p with 10 mg/kg of WP1066 or DMSO twice with a gap of one day between doses, followed by doses of 20 mg/kg every 2 days for 2 weeks. Antral tumours in 8 week untreated mice (A) and 10 week DMSO-treated vehicle controls (B) were no different in mean area, however WP1066 treatment resulted in ∼50% smaller tumours (C). This was confirmed by quantitative morphometry (D) where antral tumours were significantly smaller in WP1066 mice compared to 10 w.o. DMSO controls and 8 w.o. untreated mice (n = 7–10; p<0.05). The effect of WP1066 on cell proliferation was assessed by staining sections with an antibody for Ki-67. There was a significant decrease in the number of Ki-67 positive cells per gland in the WP1066 treated mice (F) compared to controls (E) and quantified graphically (G). The number of apoptotic cells quantified after caspase 3 staining of antral sections of a DMSO control (H) and WP1066 (I) mouse, and was quantified by counting stained cells/mm^2^ of gastric mucosa (J).

gp130^757FF^ mice develop distal stomach tumours characterised by elevated gastric IL-11 driven STAT3 activation [12], [13]. Since pJAK2 and p-STAT3 are blocked by WP1066 *in vitro*, we tested whether WP1066 would also block gastric tumour development *in vivo*. Treatment of gp130^757FF^ mice 3 times per week for 2 weeks with 10–20 mg/kg WP1066 significantly reduced gastric tumour growth by 47% from 42.11±3.21 mm^2^ to 22.36±3.64 mm^2^ (p<0.05) (Fig. 3A–D). DMSO treated tumours were no different to tumours observed in 8 week old mice, the age at which WP1066 treatment commenced, confirming that gastric tumour growth is relatively stable from 8–10 weeks [27], and that WP1066 treatment actually causes tumour regression rather than stasis (Fig. 3A–D).

To determine if proliferation was altered in gastric tumours, Ki-67 positive cells/gland were quantified in the treated cohort. WP1066 treatment significantly reduced gastric epithelial cell proliferation (DMSO 62±7.7 vs. WP1066 41.6±6.5 Ki-67 stained cells/gland, p<0.05; Fig. 3E–G) demonstrating that WP1066 can inhibit tumour growth by inhibition of cell proliferation. To assess the effects of WP1066 on tumour cell apoptosis, sections from the WP1066 or DMSO-treated cohorts were stained with an antibody against cleaved caspase 3, a marker of apoptotic cell death (Fig. 3H, I). There were significantly more apoptotic profiles in WP1066-treated tumours than controls, demonstrating a clear pro-apoptotic effect of WP1066 (WP1066 25.25±5.32 vs DMSO 2.65±0.76 stained cells/mm^2^ mucosa, p<0.05; Fig. 3J).

### WP1066 Specifically Targets JAK2 and STAT3 Phosphorylation to Suppress Gastric Tumourigenesis in gp130^757FF^ Mice

**Figure 4:**
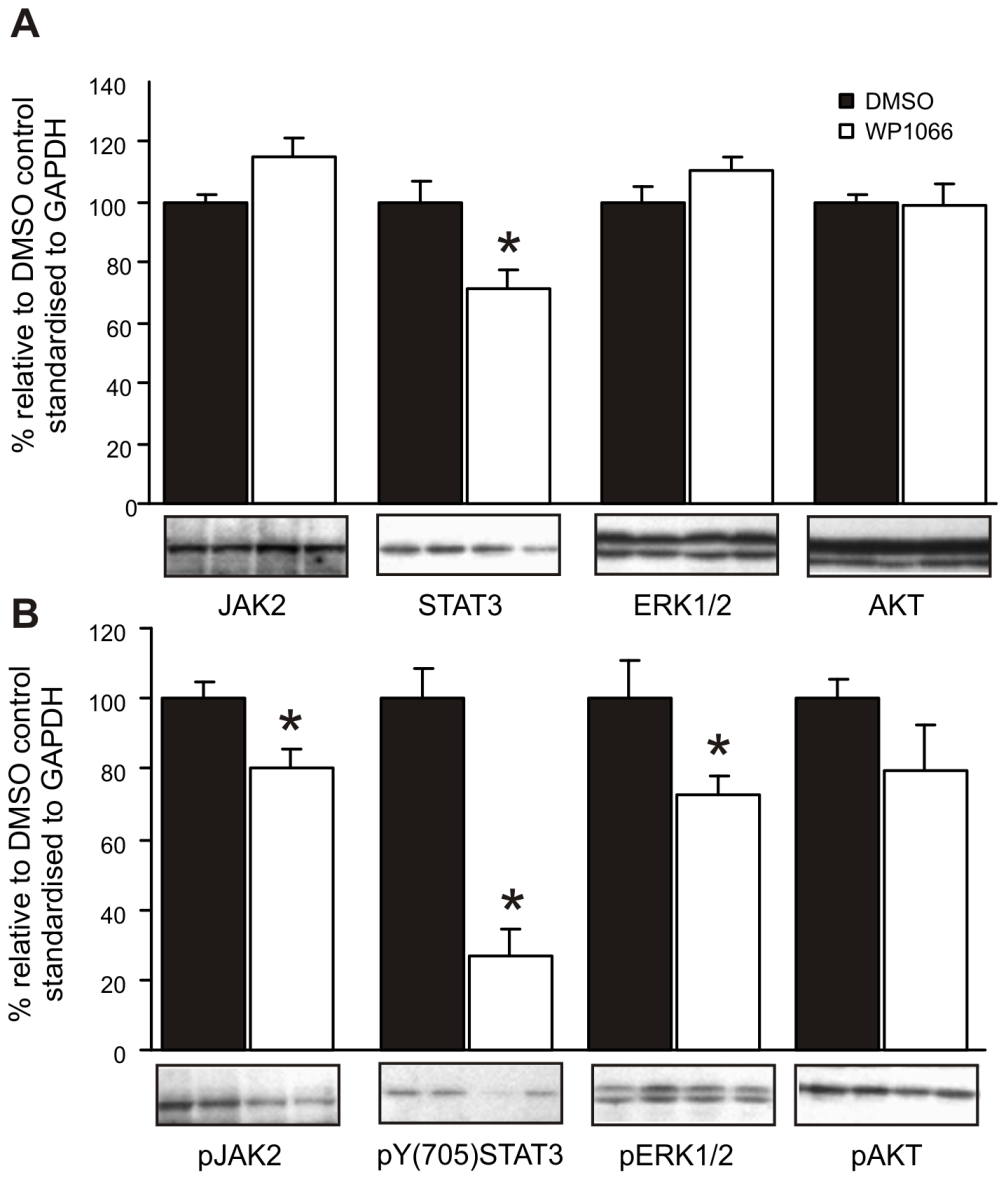
WP1066 inhibits STAT3 signalling. gp130^757FF^ mice (8 weeks old) were treated as in Fig. 3 and antral extracts quantified by Western blotting for total JAK2, STAT3, ERK1/2 and AKT (A) and pJAK2, pSTAT3, pERK1/2 and pAKT (B). Membranes were concurrently hybridised with a GAPDH antibody as a loading control. The intensity of the signal was quantified by densitometry and expressed as a percentage of the signal relative to the controls standardised to the intensity of the GAPDH signal. n = 6–10; *significantly (p<0.05).

Because gp130^757FF^ mice develop tumours in response to constitutive gp130 activation [20], we tested whether WP1066 suppressed downstream signalling pathways *in vivo.* WP1066 treatment for 2 weeks resulted in a 25% decrease in the relative amount of total STAT3 in the antral mucosa compared to DMSO treated groups (Fig. 4A). The amount of total JAK2, AKT and ERK1/2 was not changed by WP1066 treatment. This suggests that chronic treatment of gp130^757FF^ mice with WP1066 suppresses expression of STAT3.

Immunoblot analysis of signalling activation demonstrated a significant reduction in pJAK2 (80.12% ±5.70 of DMSO control), pY705 STAT3 (26.84% ±7.2 of DMSO control; Fig. 4B) and pERK1/2 (72.66% ±5.23.of DMSO control; Fig. 4B). AKT phosphorylation was not changed by WP1066 treatment. Reduced activation of JAK2 and STAT3 is consistent with the *in vitro* data and known actions of WP1066.

### WP1066 Suppressed the Inflammatory Response in the Antral Mucosa of gp130^757FF^ Mice

**Figure 5:**
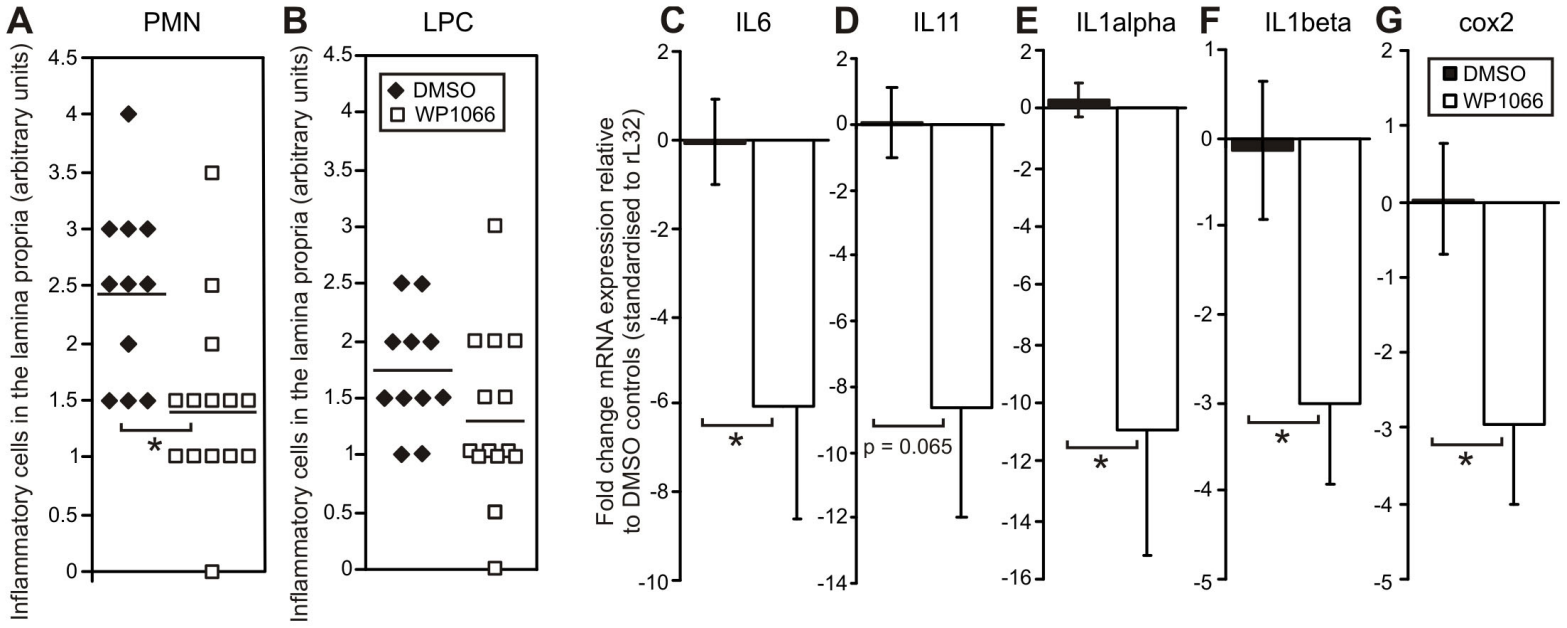
WP1066 treatment of gp130^757FF^ mice inhibits the pro-inflammatory response in the antral mucosa. gp130^757FF^ mice (8 weeks old) treated as for Fig. 3, had antral stomach tissue taken for histology and pro-inflammatory gene expression by Q-PCR after mRNA extraction. Treatment of gp130^757FF^ mice with WP1066 caused a significant reduction (p<0.05) in polymorphonuclear infiltrate (A) in the antral stomach, but lymphoplaysmocytic infiltrate was unchanged (B). Gene expression relative to the housekeeper GAPDH for IL-6 (C), IL-11 (D), IL-1 α (E), IL-1β (F) and COX-2 (G), was quantified by the ΔΔCT method. All n = 8; *p<0.05 compared to DMSO controls.

Since a chronic inflammatory response of the stomach is crucial for tumour development [27], we tested whether part of the mode of action of WP1066 is to suppress the STAT3-mediated inflammatory response, which normally contributes to tumour progression. Histological analysis revealed that polymorphonuclear infiltration into the antrum of WP1066 treated mice was significantly less than in the control mice (Fig. 5A; DMSO 2.45±0.24 vs WP1066 1.46±0.22, p<0.05), while lymphoplasmocytic infiltrate was not different between the two groups (Fig. 5B; DMSO 1.73±0.16 vs WP1066 1.32±0.20, p>0.05).

Since gastric inflammation is typically mediated by a small number of key pro-inflammatory cytokines and enzymes, we measured the expression of a select group of these by Q-PCR in DMSO and WP1066-treated stomachs. Expression of all pro-inflammatory mediators with the exception of IFNγ, TNFα and iNOS were significantly inhibited by WP1066 treatment as follows; IL-6 (Fig. 5C; 6.0±2.6 fold), IL-11 (Fig. 5D; 8.7±3.4 fold), IL-1α (Fig. 5E; 10.9±4.3) fold), IL-1β (Fig. 5F; 3±0.9 fold) and COX2 (Fig. 5G; 2.94±1.05). Therefore WP1066 inhibition of STAT3 activity and tumour progression was due in part to reduced polymophonuclear infiltration and reduced STAT3-dependent transcription of pro-inflammatory IL-6, IL-11, IL-1α, IL-1β and COX2 genes.

### WP1066 Inhibited the Expression of Amphiregulin in the Antral Mucosa of gp130^757FF^ Mice

**Figure 6:**
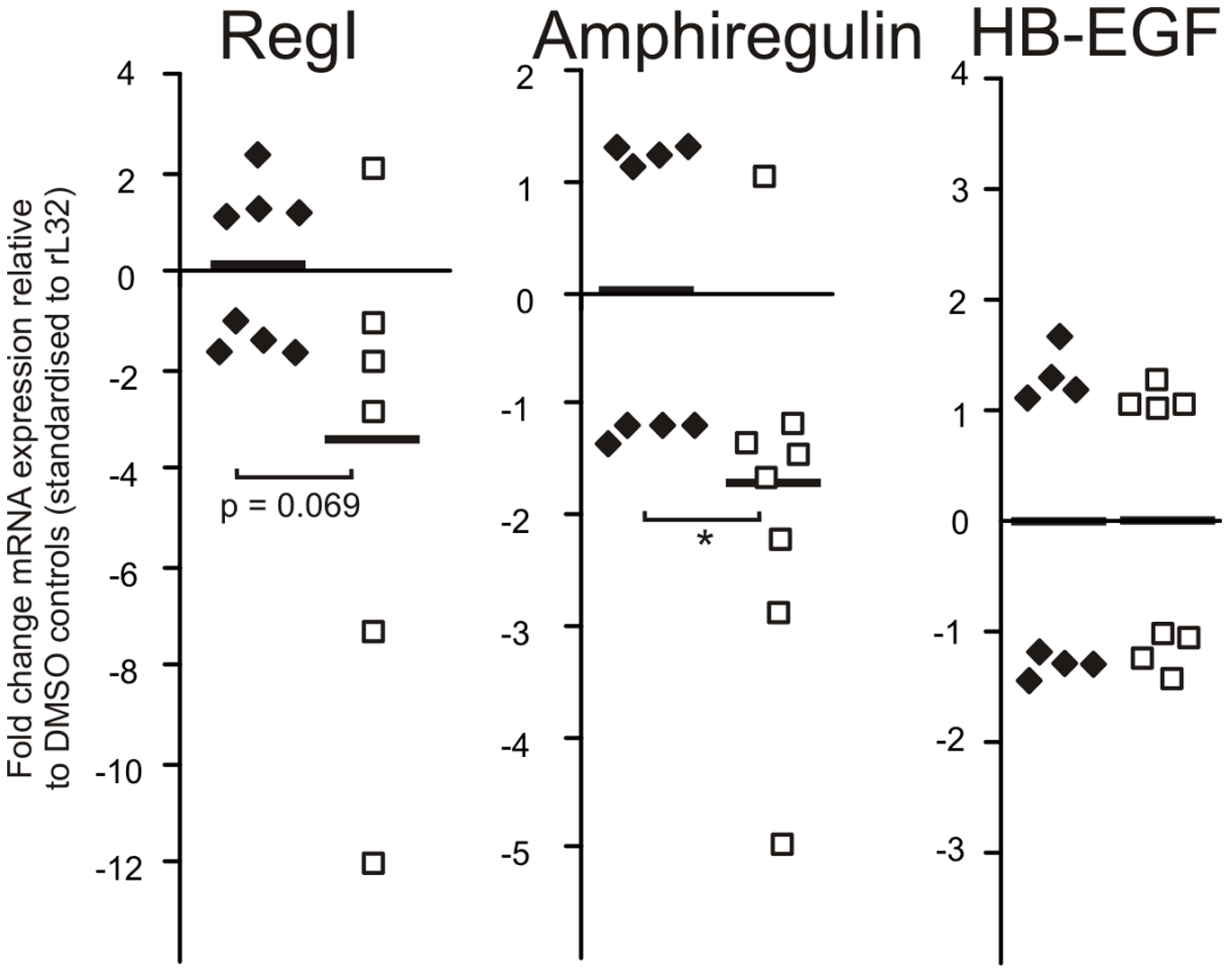
WP1066 treatment of gp130^757FF^ mice inhibits growth factor expression in the antral mucosa. gp130^757FF^ mice (8 weeks old) were treated as for Fig. 5. mRNA for Reg1, amphiregulin and HB-EGF were analysed by Q-PCR analysis and quantified by the ΔΔCT method. EGFR ligands were differentially regulated so that amphiregulin mRNA was significantly decreased in the WP1066 treated mice compared to controls (p<0.05), while HB-EGF was unchanged. The non-EGFR ligand RegI showed a strong trend towards decreased expression (p = 0.069). All n = 8; *statistically different (p≤0.05).

The effect of WP1066 treatment on the expression of growth factor ligands known to play a role in growth and differentiation of the stomach and in the development of gp130^757FF^ tumours was also assessed. WP1066 inhibited the expression of amphiregulin (1.85±0.60 fold, p<0.05), but not HB-EGF (Fig. 6) in the antral mucosa of treated mice. In addition, the stomach proliferative ligand and STAT3-regulated gene Reg1 showed a strong inhibitory trend after WP1066 treatment compared to the DMSO control antrum (Fig. 6; p = 0.069).

## Discussion

In this study we demonstrate that WP1066 dose-dependently inhibits the JAK2/STAT3 signaling pathway in human gastric cancer (AGS) cells, with a consequent 60% reduction in cell proliferation, and a smaller increase in apoptosis. The mechanism for this is likely due to the dual inhibition of phosphorylated forms of JAK2 and STAT3 thereby reducing STAT3 transcriptional activity, as has been shown for several cancer cell lines including glioma [17], [28], [29], myeloid leukemia [30], and melanoma [20]. On the other hand, WP1066 application resulted in a reciprocal increase in ERK1/2 activation coincident with increased pSHP2, the phosphatase responsible for activating the ras/MAP kinase signalling pathway. A similar observation with respect to ERK activation has been made for other cancer cell lines derived from renal carcinoma [18], and glioma [28]. Therefore, cross-regulation of STAT3 and ERK-mediated signalling downstream of IL-6 family cytokines appears to occur commonly in a ranges of tissues and cell lines [31].

WP1066 was also effective when given *in vivo*, where it blocked STAT3 activation (75%) in gp130^757FF^ mouse antral tumours, and also reduced total STAT3 protein, suggesting that it may decrease expression of the *Stat3* gene in the stomachs of treated mice. This is supported by the existence of consensus sites for activated STAT3 binding on the *Stat3* promoter, with activation of the *Stat3* gene as demonstrated using mutant *Stat3* promoter-reporter constructs and EMSA [48] or ChIP-seq [49], and suggests that WP1066 may inhibit autocrine activation of STAT3 after inhibition of JAK2/STAT3 phosphorylation.

As expected, pJAK2 levels were also reduced in the presence of WP1066 in the stomach. In contrast to the effect of WP1066 *in vitro,* ERK1/2 activation in stomachs of WP1066 treated mice was slightly decreased. The reason for this disparity may be two fold; firstly, the *in vivo* exposure to WP1066 was chronic compared to the acute exposure *in vitro*; secondly, exposure *in vivo* is more complex with multiple potential signalling pathways utilising common signal transduction molecules. Nonetheless the decreased activation of JAK2 and STAT3 is in keeping with the established role of these cytokine/growth factor signalling components in gastric cancer progression [5], [27] and demonstrates that *in vivo,* and when isolated from the effects of *H.pylori* infection, it is predominantly altered activation of STAT3, and not ERK1/2 that is crucial for tumour development.

Coincident with inhibition of STAT3 activity, application of WP1066 for only 2 weeks *in vivo* resulted in a 50% decrease in tumour area accompanied by a similar reduction in proliferation as measured by Ki-67 staining, and a concomitant increase in tumour cell apoptosis as measured by activated caspase 3 immunostaining. This inhibition of tumour growth was paralleled by a >40% decrease in polymorphonuclear cell numbers, and the up-regulation of associated pro-inflammatory cytokines including IL-1α, IL-1β as well as COX2, all of which promote gastric tumourigenesis. On the other hand WP1066 had no effect on IFNγ, TNFα, and iNOS expression commensurate with the lack of reduction in lymphocytes and macrophages (lymphoplasmocytic infiltration) over the treatment time-course. The inhibition of IL-1β after pSTAT3 inhibition by WP1066 is particularly significant, because IL-1β is an important regulator of gastric acid secretion, a potent promoter of tumour growth in a transgenic mouse model of gastric cancer [33], and an integral part of the NLRP3 inflammasome [32]. IL-11 is known to be an endogenous stimulator of antral IL-1β expression via STAT3 [13], and we have demonstrated here that IL-11 is inhibited by WP1066. Since IL-11 appears to be upstream of IL-1β in the stomach, then this is a plausible pathway for the latter’s inhibition after blockade of STAT3 activation.

The inhibitory effects of WP1066 in the gp130^757FF^ mouse model of gastric cancer, recapitulate and extend the outcomes of STAT3 haploinsufficiency generated genetically in the same mouse model [26], as well as the use of systemic antisense oligonucleotides against STAT3 [12], thus underscoring the importance of activated STAT3 signalling in facilitating gastric tumourigenesis. Given the importance of IL-11 in promoting tumour initiation and development [12], [13], and IL-6 in facilitating local polymorphonuclear infiltration [34] in the gp130^757FF^ mouse, it is significant that WP1066 effectively reduced the expression levels of both cytokines coincident with reversal of tumour growth. This supports the view that STAT3 regulates the transcription of both IL-11 and IL-6, possibly driven by autocrine feedback loops, since both cytokines preferentially utilise STAT3 transcription in the stomach [23].

Reg1 is a widely distributed member of the Reg gene family and a known gastric growth factor, which plays a prominent role as a proliferative and anti-apoptotic regulator in the stomach of both mice [26], [35] and humans [36]. We have previously shown that Reg1 is strongly induced in antral tumours of gp130^757FF^ mice coincident with IL-6 and IL-11 expression, and that haploinsufficiency of STAT3 results in reduced expression of Reg1, suggesting that it is a STAT3-regulated gene [26]. This has been confirmed in gastric cell lines where IL-11 [37] and IL-6 [36] stimulated STAT3 phosphorylation which in turn activated Reg1 transcription. Our current data are consistent with these outcomes, in that WP1066 shows a strong trend to inhibit Reg1 in gastric tumours of the gp130^757FF^ mouse along with IL-6 and IL-11 inhibition. Such inhibition would contribute to the reduced tumour burden, and increased apoptosis observed following WP1066 application in this study, and provides a partial explanation for the mechanism of action of this JAK inhibitor in the stomach.

Ligands of the epidermal growth factor receptor (EGFR) play important roles in regulating normal and carcinogenic growth throughout the gut [38]. Since we have previously shown that EGFR ligands are up-regulated at critical times during gastric tumour development in the gp130^757FF^ mouse [27], the expression and susceptibility of 2 of these ligands to blockade of STAT3 activation were measured. mRNA expression of amphiregulin but not HB-EGF, was inhibited by WP1066, suggesting that it too is regulated in a STAT3-dependent fashion, and consistent with its predominantly pro-tumourigenic actions in the stomach [35], [39]. Enhanced drive for expression of IL-6 and IL-11 may also be due to increased ligand driven EGFR activation, which also signal via STAT3, and which show increased expression during active tumour growth in the gp130^757FF^ mouse [27]. Apart from the well established autocrine regulation of amphiregulin [40] via STAT3, this growth factor can also be induced in a COX2/PGE2-dependent fashion in mouse models of gastric tumour development [41], and since COX2 was also inhibited by WP1066 then it may also contribute to the inhibition of amphiregulin.

COX2 is the rate-limiting enzyme for prostaglandin production and is induced constitutively in gastric cancer [42] by growth factors and cytokines. It is linked with the production of the inducible microsomal prostaglandin synthase (mPGES)-1, which together produce constitutively oncogenic PGE2 [41], so that transgenic mice that overexpress both COX2 and mPGES-1 develop hyperplastic gastric lesions in the stomach [43]. *H. pylori* induces both STAT3 activation [5] and COX-2 expression in gastric epithelial cells [44]. Additionally, the induction of COX2 is STAT3-mediated, since STAT3 binds the COX2 promoter, and increased COX2 positively regulates the IL-6/STAT3 axis both basally and after *H. pylori* infection [45]. COX2 can also be activated after ligand-driven nuclear translocation of the EGFR which is permissive for STAT3-dependent transcriptional activation of COX2 [46]. Together these studies and the present data suggest that expression of IL-6 family cytokines, and EGFR ligands signalling via STAT3, can induce chronic COX-2 and RegI expression. WP1066 and presumably other inhibitors of STAT3 activation can block these actions to inhibit gastric inflammation, cell proliferation and promote apoptosis and therefore block tumour development.

In summary, we have shown that WP1066 inhibits STAT3 dependent growth and blockade of apoptosis, both *in vitro* in human gastric cancer cells, and *in vivo* in an established mouse model of gastric tumourigenesis. In the latter, WP1066 caused tumours to significantly regress over the treatment timecourse, accompanied by a reduction in infiltrating inflammatory cells, pro-inflammatory cytokines, Reg1, EGFR ligand amphiregulin expression, as well as oncogenic COX2. Together this data supports efforts to further develop and apply inhibitors of JAK2/STAT3 pathway as therapeutic agents for gastric cancer treatment.
